# Regional computed tomography perfusion deficits in patients with hypoglycemia: two case reports

**DOI:** 10.1186/s42466-022-00201-z

**Published:** 2022-08-22

**Authors:** Jennifer Sartor-Pfeiffer, Mirjam Lingel, Maria-Ioanna Stefanou, Tobias Lindig, Benjamin Bender, Sven Poli, Ulf Ziemann, Andreas Fritsche, Katharina Feil, Annerose Mengel

**Affiliations:** 1 Department of Neurology and Stroke, Eberhard-Karls University of Tübingen, Tübingen, Germany; 2 Hertie-Institute for Clinical Brain Research, Eberhard-Karls University of Tübingen, Hoppe-Seyler Str.3, 72076 Tübingen, Germany; 3 Department of Diagnostic and Interventional Neuroradiology, Eberhard-Karls University of Tübingen, Tübingen, Germany; 4 Department of Internal Medicine IV, Division of Diabetology, Endocrinology and Nephrology, Eberhard-Karls University Tübingen, Tübingen, Germany; 5 Institute for Diabetes Research and Metabolic Diseases of the Helmholtz Center Munich at the University of Tübingen, Tübingen, Germany

**Keywords:** Hypoglycemia, Ischemic stroke, Stroke mimic, Hypoperfusion, Computed tomography perfusion, Type 1 diabetes mellitus, Case report

## Abstract

**Background:**

Hypoglycemia in patients with diabetes mellitus, particularly type 1 can mimic acute ischemic stroke by causing focal neurological deficits. In acute ischemic stroke, the interpretation of emergency imaging including computed tomography with angiography and perfusion is crucial to guide revascularizing therapy including intravenous thrombolysis. However, different metabolic abnormalities and stroke mimics can cause focal hypoperfusion.

**Methods:**

We describe two type 1 diabetes patients presenting with acute focal neurological deficits and hypoglycemia, who underwent multimodal computed tomography and follow-up imaging.

**Case presentation:**

Patient 1, a 20-year-old man presented with aphasia and interstitial glucose level of 54 mg/dl. Patient 2, a 77-year-old man presented with aphasia, mild right-sided brachiofacial paresis and interstitial glucose level of 83 mg/dl. On brain imaging, no acute infarct signs were noted. Yet, both had focal left hemispheric cerebral hypoperfusion without large-vessel occlusion or stenosis. Due to persistent symptoms after normalization of blood glucose and despite a perfusion imaging pattern that was interpretated as non-typical for ischemia, both patients underwent thrombolysis without any complications.

**Conclusion:**

Computed tomography perfusion might help to discriminate hypoglycemia with focal neurological signs from acute stroke, but further evidence is needed.

Dear Prof. Hacke,

Hypoglycemia is feared in type 1 diabetes mellitus (T1DM) and can manifest with focal neurological signs (HFNS) [1] as stroke mimic (SM). In acute ischemic stroke (AIS), interpretation of emergency multimodal computed tomography (CT) is crucial to provide intravenous thrombolysis (IVT). Especially CT perfusion (CTP) could help to differentiate AIS from HFNS [2].

Focal transient lesions in cerebral diffusion weighted (DWI) magnetic resonance imaging (MRI) have been described in HFNS [1]. Focal hypoperfusion in CTP in conjunction with HFNS has been previously reported once [3].

We describe two patients with T1DM and initial hypoglycemia receiving IVT due to persistent neurological deficits despite normalization of blood glucose levels (BGL). Multimodal CT on admission and follow-up non-contrast CT (ncCT) were performed in both, and 1.5 Tesla MRI in patient 1. CTP was performed as part of standard clinical practice protocol. In CTP, salvageable tissue was calculated based on mismatch ratio as follows: hypoperfusion area with Tmax > 6 s (tissue at risk) minus ischemic core with cerebral blood flow (CBF) < 30% [4]. Volume of hypoperfusion was measured manually based on CBF for both patients and time-to-peak (TTP) in patient 2 (or time-to-drain as most relevant in patient 1) and calculated with ABC/2-formula [5].

## Case 1

**Fig. 1:**
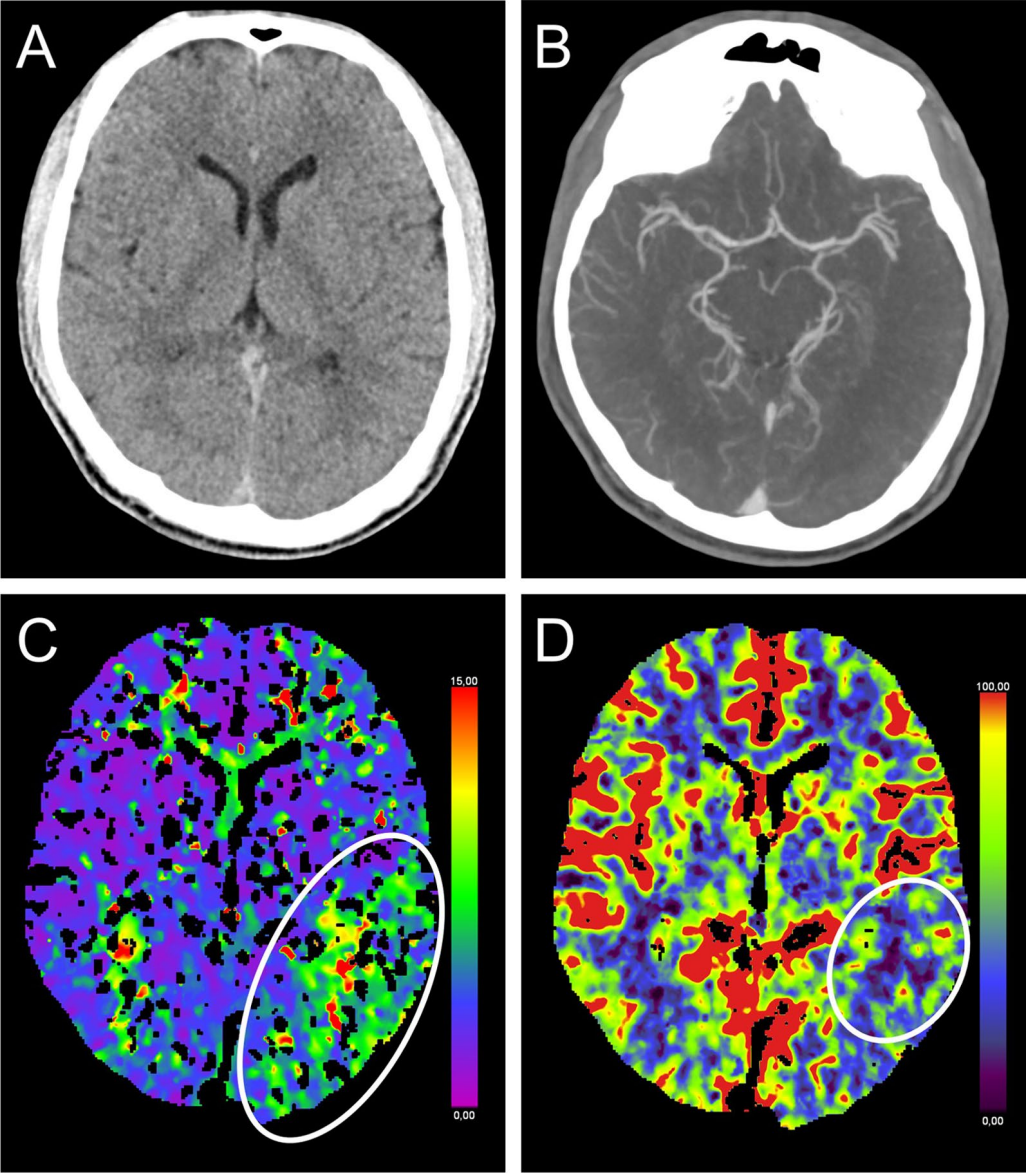
NcCT, CTA, and CTP of patient 1. Exemplary cuts of first ncCT (**A**), CTA (**B**), and CTP (**C**–**D**) 87 min after onset of aphasia. CTA without relevant stenosis of extra- or intracranial vessels (**B**). Follow-up ncCT 31 h after onset (not shown) did not show infarct demarcation compared to first ncCT (**A**). Hypoperfusion parieto-temporal on TTD (**C**) and smaller reduced perfusion on CBF (**D**) marked with circle. Only mild delay (2 s) was detected in corresponding TTP. CBF, cerebral blood flow; CT, computed tomography; CTA, CT angiography; CTP, CT perfusion; ncCT, non-contrast CT; TTD, time-to-drain; and TTP, time-to-peak

A 20-year-old man with T1DM woke up with headache and dizziness, measured interstitial glucose level (IGL) of 54 mg/dl, and took glucose orally. Two hours later he woke up with aphasia. With BGL of 81 mg/dl, emergency medical physicians administered intravenous glucose. Examination on admission (BGL of 129 mg/dl) revealed moderate fluent aphasia. CTP showed focal hypoperfusion left parieto-temporally (Tmax 2.6 s on TTP) (Fig. 1) [4, 5] without large-vessel occlusion or stenosis in CT angiography (CTA). Due to persisting symptoms > 60 min after normalized BGL, patient received IVT 110 min after onset. The next day, he was asymptomatic. MRI after 53 h revealed punctual and transient cortical DWI lesion without apparent diffusion coefficient (ADC) reduction left parietally.

## Case 2

**Fig. 2:**
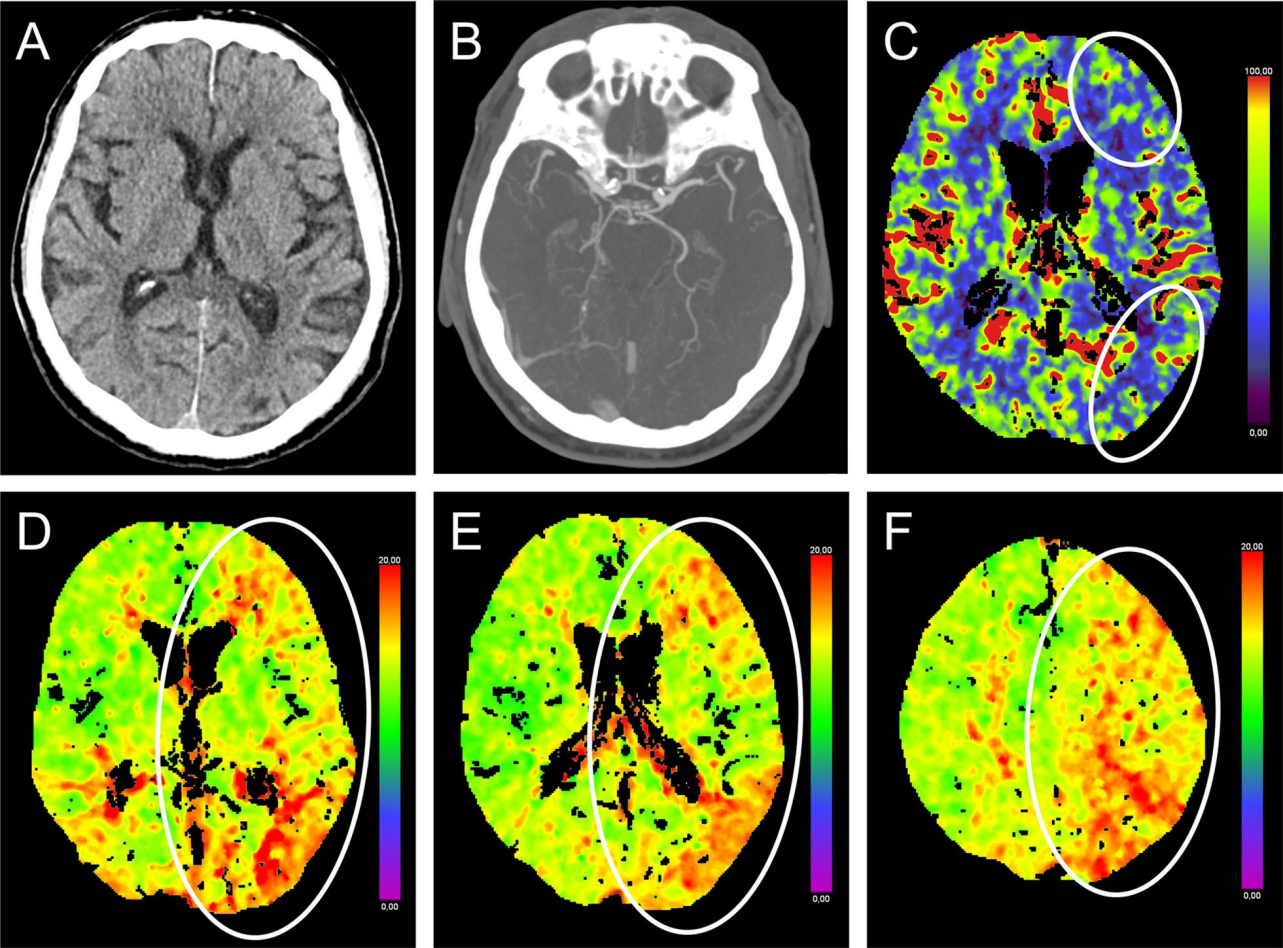
NcCT, CTA, and CTP of patient 2. Exemplary cuts of first ncCT (**A**), CTA (**B**), and CTP (**C**–**F**) 70 min after onset of symptoms. CTA without relevant stenosis but with elongation of extra- or intracranial vessels (**B**). Follow-up ncCT 24 h after onset (not shown) did not show infarct demarcation compared to first ncCT (**A**). Hypoperfusion in left frontal and left parieto-temporo-occipital areas on TTP with Tmax of > 6 s (**D**–**F**) and smaller reduced perfusion on CBF (**C**) marked with circle. CBF, cerebral blood flow; CT, computed tomography; CTA, CT Angiography; CTP, CT Perfusion; ncCT, non-contrast CT; Tmax, time-to-maximum; and TTP, time-to-peak

A 77-year-old man with T1DM was found with acute aphasia. Attributing his symptoms to hypoglycemia, he took glucose orally (IGL of 83 mg/dl). Several comorbidities, including coronary heart disease, ischemic cardiomyopathy, and implanted non-MRI-conditional pacemaker were known. Upon admission, examination revealed non-fluent aphasia and mild right-sided brachiofacial paresis. Admission BGL was 83 mg/dl 50 min after onset. NcCT showed chronic right cerebellar ischemic defect. CTP showed hypoperfusion left frontal and parieto-temporo-occipitally (Tmax 2.6 s on TTP) [4, 5] without large-vessel occlusion or stenosis in CTA (Fig. 2). With persisting symptoms > 30 min after normalized BGL and hypoperfusion on TTP, patient 2 received IVT 82 min after onset. Forty-three hours later, he was asymptomatic. NcCT after 24 h excluded demarcation.

## Discussion

SM pose diagnostic challenges and especially HFNS requires neurologic co-assessment. MRI is first choice modality in differentiating SM from AIS, but often not available 24/7. CT is often used as primary diagnostic modality and CTP is known for high diagnostic accuracy in predicting mismatch between infarct core and salvageable tissue in AIS [6]. CTP can facilitate the early differentiation of AIS from SM [2, 7, 8], including migraine [7] or seizures [8]. In diabetic patients, focal lesions on DWI MRI following HFNS have been previously detected [1].

Neuroglycopenic symptoms usually manifest below a threshold of 70 mg/dl, which shifts to higher threshold in poorly controlled T1DM [9], also found in our patients based on HbA1c-findings (HbA1c 9.1% in patient 1, 9.6% in patient 2), possibly resulting in neuroglycopenic symptoms *above* 70 mg/dl.

In patient 1, we found a transient punctual DWI lesion without ischemia-typical ADC decrease, which topologically did not explain aphasia. Together with patient’s young age, AIS must be questioned discussing symptoms as HFNS/SM. In patient 2, symptoms persisted > 24 h, but without demarcation in ncCT. Considering patient's high vascular risk profile and chronic cerebellar infarct, AIS seems possible. We excluded status epilepticus as differential diagnosis in both cases due to EEG with focal left hemispheric slowing but without epileptic potentials [10], while the patients were still symptomatic.

Concerning diagnosis, time course of neurological manifestations with acute symptom onset, persisting deficits despite normalized BGL, and slow symptom recovery following IVT, support cerebrovascular origin. Yet, hypoperfusion could not be fully aligned with vascular territories and was finally considered as non-typical for AIS [4]. Thus, we cannot clearly differentiate between hypoperfusion due to hypoglycemia or AIS in our cases. Other important differential diagnoses should also be considered, including migraine [7] or status epilepticus [8, 10], and early EEG should be conducted in every patient.

Clinical awareness of regional hypoperfusion in T1DM patients with hypoglycemia is crucial. If neurologic deficits persist despite restoration of normoglycemia, emergency physicians should consider AIS and not postpone recanalization therapy if necessary. Further studies to clarify if CTP can help distinguishing HFNS from AIS are necessary.

## Data Availability

The datasets used and/or analyzed during the current study are available from the corresponding author on reasonable request.

## Abbreviations

ADC: Apparent diffusion coefficient
AIS: Acute ischemic stroke
BGL: Blood glucose level
CBF: Cerebral blood flow
CT: Computed tomography
CTA: Computed tomography angiography
CTP: Computed tomography perfusion
DWI: Diffusion weighted images
HFNS: Hypoglycemia with focal neurological signs
IGL: Interstitial glucose level
IVT: Intravenous thrombolysis
MRI: Magnetic resonance imaging
T1DM: Type 1 diabetes mellitus
ncCT: Non-contrast CT
SM: Stroke mimic
Tmax: Time-to-maximum
TTP: Time-to-peak
